# Effect of Storage Conditions on the Thermal Stability and Crystallization Behaviors of Poly(L-Lactide)/Poly(D-Lactide)

**DOI:** 10.3390/polym13020238

**Published:** 2021-01-12

**Authors:** Tien-Wei Shyr, Huan-Chieh Ko, Tzong-Ming Wu, Meifang Zhu

**Affiliations:** 1 Department of Fiber and Composite Materials, Feng Chia University, Taichung 407802, Taiwan; hcko@mail.fcu.edu.tw; 2 National Synchrotron Radiation Research Center, Hsinchu 30076, Taiwan; 3 Department of Materials Engineering, National Chung Hsing University, Taichung 407802, Taiwan; tmwu@dragon.nchu.edu.tw; 4 State Key Laboratory for Modification of Chemical Fibers and Polymer Materials, Donghua University, Shanghai 201620, China; zmf@dhu.edu.cn

**Keywords:** polylactide, stereocomplex, crystallization, storage condition

## Abstract

Polylactide (PLA) is a biodegradable thermoplastic aliphatic polyester. The thermal stability and crystallization behavior of PLA are extremely sensitive to storage, processing, and usage conditions. This work systematically studied the thermal stability and crystallization behavior of poly(L-lactide) (PLLA), poly(D-lactide) (PDLA), and a PLLA/PDLA (LD) blend, which were stored under two sets of laboratory storage conditions: (1) stored in a vacuum-free desiccator and (2) stored in vacuum-sealed bags. Both were stored at room temperature for 3 years. Gel permeation chromatography results revealed that the PLLA, PDLA, and LD samples hydrolyzed slowly when stored in vacuum-sealed bags and degraded significantly when stored in a vacuum-free desiccator; this process significantly reduced the thermal stability of the samples stored in the vacuum-free desiccator. Owing to hydrolysis, the levorotation and dextrorotation (L- and D-) molecular chains were shortened; consequently, more nuclei were formed, and this caused the melting points of the PLLA, PDLA, and LD samples to decrease and the melting enthalpy of the crystals in these samples to increase. Wide-angle X-ray diffraction analysis revealed that when the L- and D- molecular chains were packed side by side to form stereocomplex crystals and the randomly arranged L- and D- molecular chains were easy hydrolyzed and degraded, this interfered with the formation of homocrystals in LD. When PLLA, PDLA, and LD samples are stored in a vacuum-free desiccator, they will be significantly hydrolyzed, resulting in the formation of only stereocomplex crystals, and no homocrystals are observed.

## 1. Introduction

Polylactide (PLA) is a biodegradable thermoplastic aliphatic polyester; its ester backbone is susceptible to chemical hydrolysis in aqueous environments [1]. This characteristic of PLA is of interest with respect to materials that require biodegradable applications [2]; however, it is also a main problem when storing and processing these materials. The degradation of PLA is primarily due to the hydrolysis of the ester linkages, which occur more or less randomly along the polymer backbone. The presence of weakly hydrolyzed bonds makes PLA sensitive to moisture and heat. Moisture will promote the hydrolysis of PLA due to the cleavage of the –C–O– ester bond [3,4,5]; thus, the properties of PLA, especially their mechanical and rheological properties [6], are extremely sensitive to storage, processing, and usage conditions [7,8,9]. For example, the following storage conditions are recommended for crystalline lactide: storage at ambient temperature for 1 year and at 4 °C throughout one year in airtight bags and vapor barriers (including an inner plastic bag and outer aluminum pouch). Crystalline lactide may be oxidized by specific components (e.g., moisture, oxygen, and acid contamination) in the environmental atmosphere and therefore may rapidly decompose in air. The rate of lactide decomposition depends on the contact surface and temperature [10]. The formation of homocrystal and stereocomplex crystal in chips and fibers of poly(L-lactide) (PLLA) and poly(D-lactide) (PDLA) of varying weight ratio has been investigated [11,12]. Because PLA can be hydrolyzed to obtain lactic acid under biological conditions, potential biomedical applications are concerned with the difference in hydrolytic activity between stereocomplex crystals with β-form 3_1_-helices and homocrystals with α-form 10_3_-helices. Li and Vert [13] demonstrated that stereocomplex crystals formed during the hydrolysis of poly(L-lactide-co-D-lactide) (L/D ratios of 62.5/37.5; intrinsically amorphous) films in a phosphate buffer solution. The relatively random monomer unit sequences without the addition of stereocomplex crystals produced greater hydrolysis. On the other hand, poly(L-lactide-co-D-lactide) with L/D ratios of 62.5/37.5 formed stereocomplex crystals after long-term hydrolysis [14]. Owing to the stereoselective interaction between PLLA and PDLA chains, stereocomplex crystals with a dense crystal structure exhibit hydrolysis resistance superior to that of homocrystals; therefore, homocrystals become easily detached from the surface during the etching process [15,16,17]. Multiple studies have investigated the hydrolytic degradation of well stereocomplex blends of PLLA/PDLA and nonblended films prepared with solvent casting technology [18,19,20,21,22]; these studies have reported that a well stereocomplex 1:1 blended film is more resistant to hydrolysis than a nonblended film is, because of the strong interaction between L- and D-lactide unit sequences and the solid three-dimensional micronetwork formed after stereocomplexation [18]. The activation energy for the degradation of stereocomplex crystals (97.3 kJ mol^−1^) is considerable higher than that required for the degradation of PLLA α-form crystals (75.2 kJ mol^−1^) [21]. The hydrolysis of samples stored under different storage conditions is significantly different, and the thermal stability and crystallization behavior of PLA are extremely sensitive to storage, processing, and usage conditions. Therefore, this study systematically studied the thermal stability and crystallization behavior of poly(L-lactide) (PLLA), poly(D-lactide) (PDLA), and a PLLA/PDLA (LD) blend, which were stored under two sets of laboratory storage conditions: (1) stored in a vacuum-free desiccator and (2) stored in vacuum-sealed bags. Both were stored at room temperature for 3 years. Thermogravimetric analysis (TGA), gas chromatography–mass spectrometry (GC–MS), gel permeation chromatography (GPC), differential scanning calorimetry (DSC), and wide-angle X-ray diffraction (WAXD) were employed to investigate the homocrystallization and stereocomplex crystallization of LD after storage under both sets of conditions.

## 2. Materials and Methods

PLLA (Synterra^®^ PLLA 1510) and PDLA (Synterra^®^ PDLA 1010) were supplied in chip form by Synbra Technology BV (Etten-Leur, Netherlands), the residual monomer of PLLA was less than 1% and the L-Isomer of PDLA was less than 1%. The initial thermal degradation temperatures of the received PLLA and PDLA were 300 °C, as measured using TGA. LD was prepared by melt-blending in a mixer at 190–220 °C with mechanical stirring at 100 rpm and a throughput rate of 3.5 kg/h, with a PLLA:PDLA feed ratio (by weight) of 50:50. As mentioned, PLLA, PDLA, and as-blended LD were stored under two sets of conditions, both of which are common in most polymer laboratories. The duration of the experimental storage was 3 years. The sets of storage conditions are described as follows:

1. Storage in a zipper bag in a vacuum-free desiccator containing silica gel at a room temperature of 25 ± 3 °C and 50% relative humidity.
2. Storage in a vacuum-sealed bag at a room temperature of 25 ± 3 °C.

The received PLLA, PDLA, and as-blended LD samples used in this study were denoted as PLLA-R, PDLA-R, and LD-R, respectively. The PLLA-R, PDLA-R, and LD-R samples stored in zipper bags in a vacuum-free desiccator were denoted as PLLA-D, PDLA-D, and LD-D, respectively, and those stored in vacuum-sealed bags were denoted as PLLA-V, PDLA-V, and LD-V, respectively.

The thermal degradation behavior of each sample was characterized through TGA (TGA2050, Du-Pont, Delaware, DE, USA) with a thermal analysis system (TA2000, Delaware, DE, USA). The instrument was calibrated with Ni. The sample was heated from room temperature to 600 °C at a rate of 10 °C/min. Dry nitrogen was used as the purge gas at a rate of 100 cm^3^/min.

Degradation products were analyzed using a Mettler-Toledo Analytical instrument (2-HT, Novate Milanese, Italy), which was coupled to a gas chromatograph (7890A, Agilent Technologies, Madrid, Spain) equipped with a mass selective detector (5975, Agilent Technologies, Madrid, Spain). The column used for analysis was a 30 m-long HP-5 (0.25 mm thickness), using helium as a carrier gas, and the split ratio was 50:1. The GC oven was programmed at 40 °C for 5 min, then gradually increased at 10 °C min^−1^ to 280 °C, and held for 5 min. The mass selective detector was programmed to detect masses between 1.6 and 1050 amu. PLA samples were pyrolyzed at 180 °C for 0.5 s. The identification of PLA degradation products was confirmed by the characteristic fragment patterns observed in GC/MS spectra and compared with the literature mass spectra.

The crystallization and melting behaviors were observed through DSC (DSC Q10, Du-Pont) with a thermal analysis system (TA2000). The instrument was calibrated with In and Pb. The flow rate of the purge gas, N_2_, was approximately 50 cm^3^/min.

The number-average molecular weight (*M*_n_), weight-average molecular weight (*M*_w_), and intrinsic viscosity (I.V.) of the sample were measured using the Viscotek GPC System (1122 pump, 2707 Auto-Injector, 270 LS Laser Light Scattering Detector/Viscometer, Shodex 71 RI Detector, OmniSEC 4.6 Station, Malvern, United Kingdom) with an HFIP 806M Shodex column. The sample was dissolved in hexafluoroisopropanol (HFIP) for 12 h and then filtered through a 0.2 μm polytetrafluoroethylene filter membrane. The oven temperature, flow rate, and analysis time were set at 30 °C, 1 mL∙min^−1^ with HFIP, and 60 min, respectively.

The crystal structure was obtained using a wide-angle X-ray diffractometer (D8 Discover, Bruker) with Cu Kα radiation. The electric voltage and current were 50 kV and 1000 μA, respectively. The 2θ scanning angle was between 10° and 30° with a scan step size of 0.02° and a scan step time of 0.5 s.

## 3. Results and Discussion

**Figure 1:**
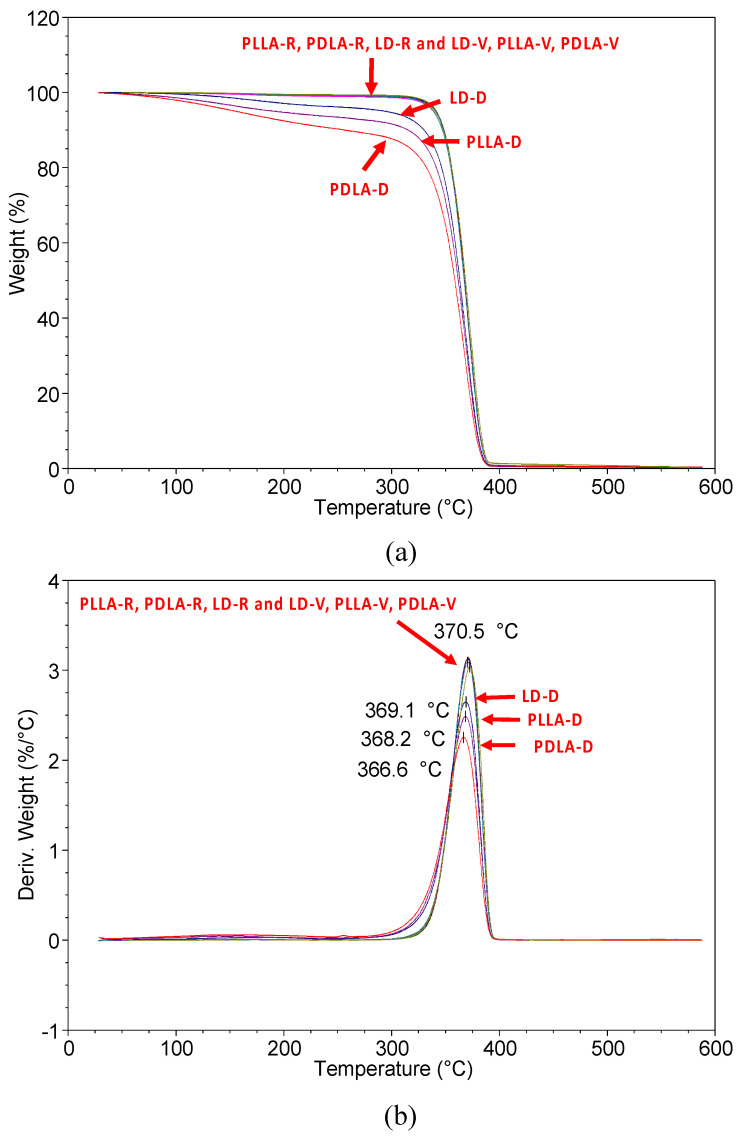
(**a**) Thermogravimetric analysis (TGA) and (**b**) derivative thermal gravimetric (DTG) curves of poly(L-lactide) (PLLA), poly(D-lactide) (PDLA), and the PLLA/PDLA (LD) blend at a heating rate of 10 °C/min.

**Figure 2:**
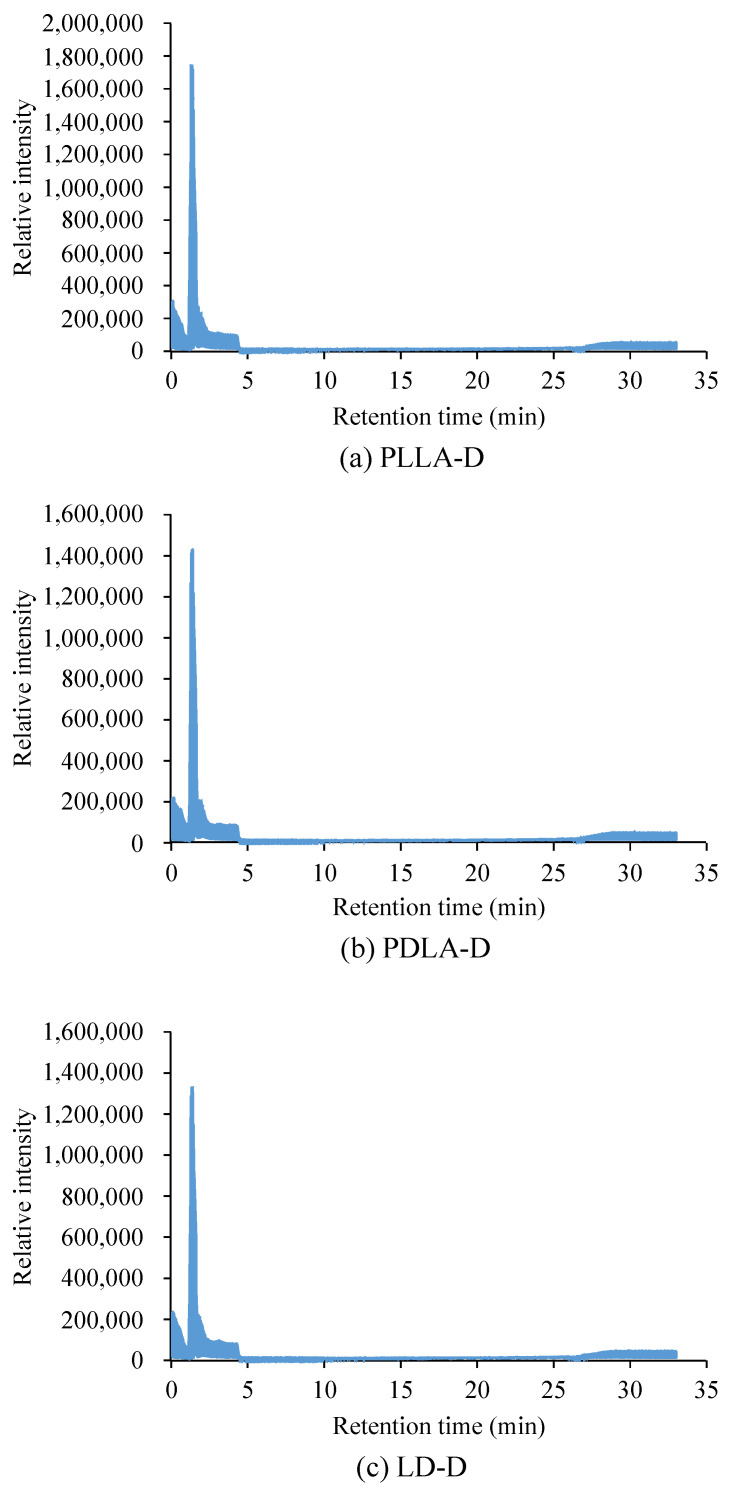
GC–MS chromatogram of (**a**) PLLA-D, (**b**) PDLA-D, and (**c**) LD-D pyrolyzed for 0.5 s at 180 °C.

**Figure 3:**
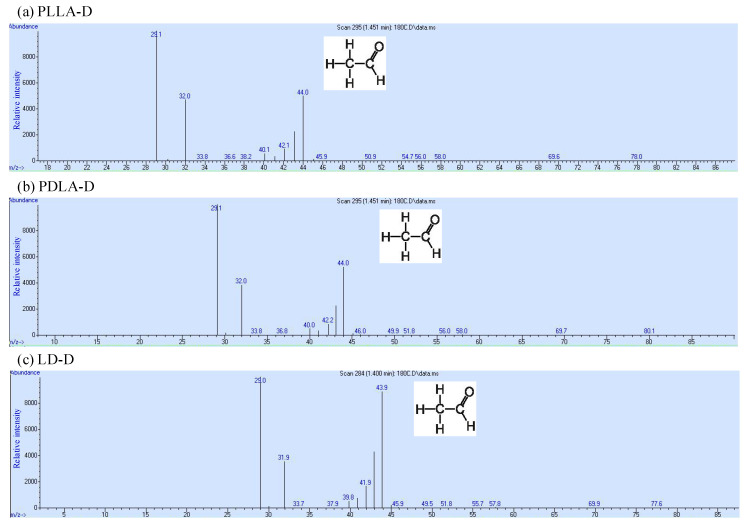
GC–MS characteristic fragmentation patterns of (**a**) PLLA-D, (**b**) PDLA-D, and (**c**) LD-D of peak 1.4 min.

The thermal stability of PLLA, PDLA, and LD under the two sets of storage conditions is shown in Figure 1. PLLA-R, PDLA-R, LD-R, PLLA-V, PDLA-V, and LD-V had onset temperatures (*T*_0_) of thermal degradation of 300 °C and a maximum degradation rate (*T*_max_) of 370~371 °C. The *T*_0_ values of PLLA-D, PDLA-D, and LD-D were 89, 64, and 130 °C, respectively, and their *T*_max_ values were 368, 367, and 369 °C, respectively. The thermal stability of PLLA-R, PDLA-R, LD-R, PLLA-V, PDLA-V, and LD-V were almost identical, whereas those of PLLA-D, PDLA-D, and LD-D were markedly lower; LD-D had the highest thermal stability, followed by PLLA-D and then PDLA-D. The samples stored in the vacuum-free desiccator exhibited significant levels of hydrolytic degradation, leading to a significant reduction in the *T*_0_ and *T*_max_ values of PLLA-D, PDLA-D, and LD-D; this finding indicates that storage conditions can significantly affect the thermal stability of PLA. According to the *T*_0_ of the sample, the degradation products of PLLA-D, PDLA-D and LD-D were analyzed using gas chromatography–mass spectrometry at 180 °C. Each degradation product was detected and identified by the mass spectra of each main chromatographic peak (see Figure 2). There was a peak at 1.4 min, and the mass-to-charge ratio (*m/z*) was 44. The characteristic fragmentation patterns observed in the Py–GC/MS spectra are shown in Figure 3. McNeill and Leiper studied the degradation of PLLA under controlled heating and isothermal conditions and reported that the main products were cyclic oligomers, including lactide and other low-boiling products such as carbon dioxide, acetaldehyde, ketene, and carbon monoxide [23,24]. After the library search and literature mass spectra analysis, PLLA-D, PDLA-D and LD-D degradation products were identified, and the main degradation product was found to be acetaldehyde [25,26]. The results showed that all samples had the same main degradation products at 180 °C.

**Figure 4:**
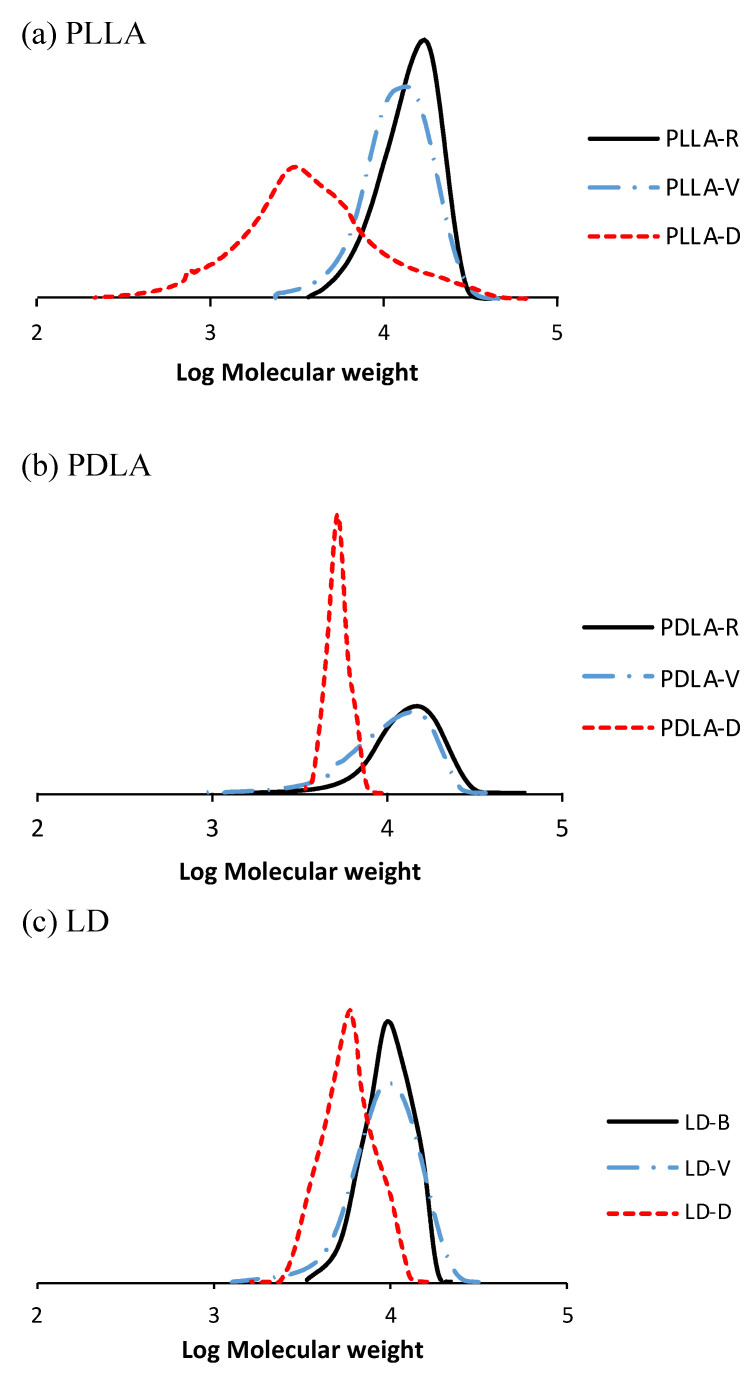
Gel permeation chromatography (GPC) curves of (**a**) PLLA, (**b**) PDLA, and (**c**) LD.

**Figure 5:**
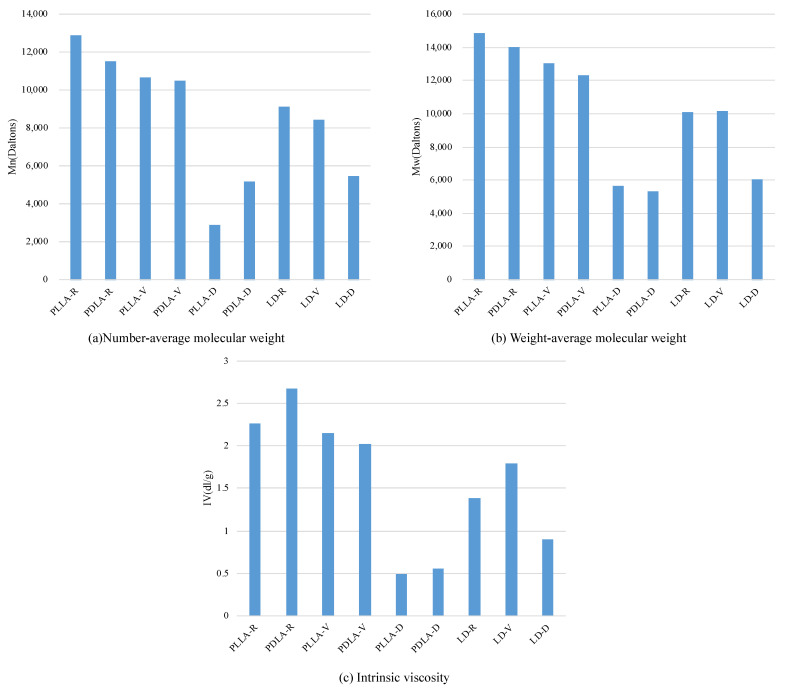
(**a**) *M*_n_; (**b**) *M*_w_; and (**c**) intrinsic viscosity (I.V.) of PLLA, PDLA, and LD.

Figure 4 demonstrates the GPC curves of the PLLA, PDLA, and LD samples under the two sets of storage conditions. The molecular weight distributions of all the samples shifted to lower molecular weights during storage. The *M*_n_, *M*_w_, and I.V. of PLLA, PDLA, and LD obtained through GPC are plotted in Figure 5. The results of *M*_n_, *M*_w_, and I.V. revealed that the samples stored in vacuum-sealed bags hydrolyzed slowly, whereas those stored in the vacuum-free desiccator degraded significantly. This indicates that the hydrolysis levels of PLLA, PDLA, and LD samples under two different storage conditions, both of which are common in most polymer laboratories, are quite different. Although the molecular weight of LD-R is lower than that of PLLA-R and PDLA-R before hydrolytic degradation, the molecular weights of LD-D were significantly higher than that of PLLA-D and PDLA-D after hydrolytic degradation. In addition, the thermal stability of LD-D was higher than that of PLLA-D and PDLA-D (Figure 1). These results indicate that stereocomplexation strongly prevented hydrolytic degradation, which have been reported in the literature [18,19,20,21,22].

**Figure 6:**
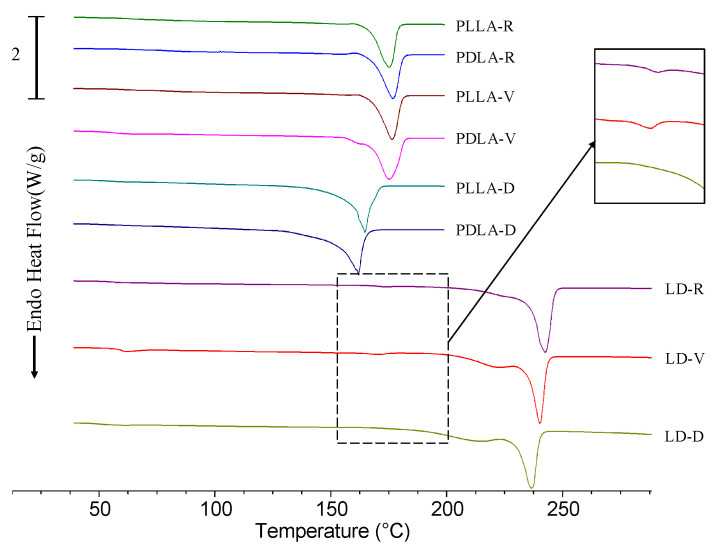
First differential scanning calorimetry (DSC) heating curves of PLLA and PDLA from 30 to 200 °C and those of the LD blend from 30 to 290 °C. The heating rate was 10 °C/min.

**Figure 7:**
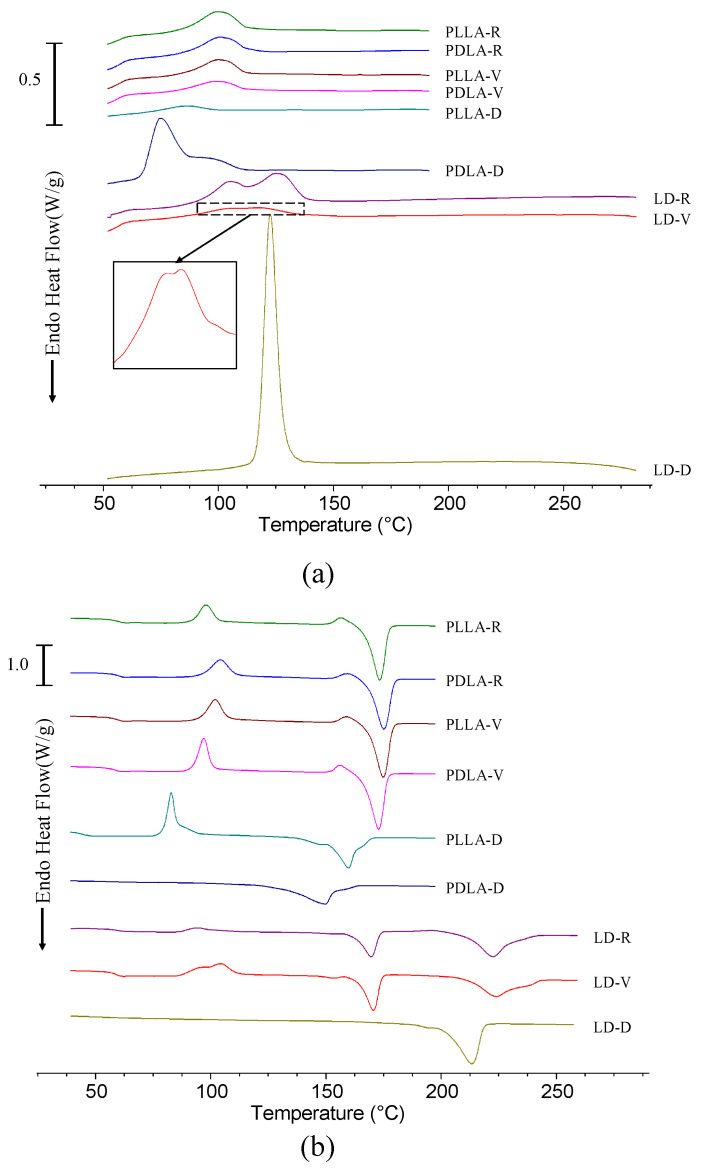
DSC (**a**) cooling curves and (**b**) reheating curves for PLLA, PDLA, and LD at a heating rate of 10 °C/min.

The melting behaviors of PLLA, PDLA, and LD are illustrated in Figure 6. The first heating curves of PLLA-V and PDLA-V revealed melting behaviors almost identical to those of PLLA-R and PDLA-R. These heating curves had a melting peak at 175–177 °C; these peaks were caused by the melting of homocrystals [27]. The corresponding cooling and reheating curves of PLLA and PDLA at 10 °C/min after melting at 200 °C for 3 min are shown in Figure 7. PLLA-V and PDLA-V exhibited homocrystallization behaviors almost identical to those of PLLA-R and PDLA-R during cooling (Figure 7a). They demonstrated cold crystallization peaks followed by melting peaks at 173–175 °C during reheating owing to the melting of homocrystals. The melting enthalpy values in the reheating curves of PLLA-V and PDLA-V were 51.3 and 52.5 J/g, respectively (Figure 7b). These values were slightly higher than the corresponding values for PLLA-R and PDLA-R, which were 50.7 and 50.4 J/g, respectively. However, the melting behaviors of PLLA-D and PDLA-D differed significantly from those of PLLA-R and PDLA-R; the melting peaks of the first heating curves of PLLA-D and PDLA-D occurred at 164.7 and 161.8 °C, respectively (Figure 6). The cooling curve of PDLA-D indicated high crystallization enthalpy (Figure 7a). During reheating, the melting peaks of PLLA-D and PDLA-D occurred at 159.8 and 149.3 °C, respectively (Figure 7b). No cold crystallization peak of PDLA-D was observed during reheating. The melting enthalpy values in the reheating curves of PLLA-D and PDLA-D were 52.1 and 54.0 J/g, respectively. Evidently, the melting peaks of PLLA-D and PDLA-D were significantly lower than those of PLLA-R and PDLA-R. However, the melting enthalpy values of PLLA-D and PDLA-D were higher than the corresponding values of PLLA-R and PDLA-R.

**Figure 8:**
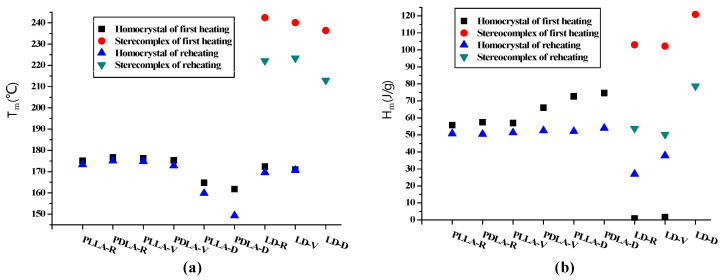
(**a**) Melting peaks and (**b**) melting enthalpy values of the first heating and reheating curves for PLLA, PDLA, and LD at a heating rate of 10 °C/min.

During the first heating, the melting and crystallization behaviors of LD-V were almost identical to those of LD-R (Figure 6). LD-V had a low melting peak at 170.9 °C with a melting enthalpy value of 1.7 J/g owing to homocrystals as well as a melting peak at 240.1 °C with a melting enthalpy value of 102.2 J/g owing to stereocomplex crystals [27]. The melting enthalpy of LD-V stereocomplex crystals was almost identical to that of the LD-R stereocomplex crystals: approximately 103.0 J/g (Figure 8). The cooling curves of LD-R and LD-V exhibited double crystallization peaks, which were caused by homocrystallization at 102–105 °C and stereocomplex crystallization at 117–124 °C (Figure 7a). The crystallization enthalpy value of LD-V (15.9 J/g) was lower than that of LD-R (37.1 J/g) during cooling. The reheating curves of LD-R and LD-V exhibited cold crystallization peaks followed by double melting peaks (Figure 7b). The lower melting peak temperature resulted from the melting of homocrystals at 169–171 °C, whereas the higher melting peak temperature resulted from the melting of stereocomplex crystals at 222–224 °C. The melting enthalpy values resulting from the homocrystals of LD-R and LD-V were 27.0 and 37.8 J/g, respectively, and the melting enthalpy values resulting from the stereocomplex crystals of LD-R and LD-V were 53.8 and 50.3 J/g, respectively (Figure 8).

The melting behavior of LD-D differed significantly from that of LD-R. During the first heating process, LD-D exhibited a melting peak at 236.4 °C due to the melting of stereocomplex crystals (Figure 6) [27]. No homocrystals were observed. LD-D exhibited high crystallization enthalpy in its cooling curve and only one melting peak during reheating; these observations resulted from the crystallization and melting of the stereocomplex crystals (Figure 7). The melting peak and melting enthalpy values of LD-D were 213.0 °C and 78.8 J/g, respectively (Figure 8). Homocrystallization did not occur in LD-D.

Homocrystals were observed in PLLA-R, PDLA-R, PLLA-V, PDLA-V, PLLA-D, PDLA-D, LD-R, and LD-V during the first heating, cooling, and reheating processes. Compared with the thermal behaviors of the received PLLA-R, PDLA-R, and as-blended LD-R samples, no significant differences were observed in the corresponding behaviors of the PLLA-V, PDLA-V, and LD-V samples stored in vacuum-sealed bags. In contrast, the cooling and reheating curves indicated that the samples stored in the vacuum-free desiccator exhibited significant hydrolytic degradation; specifically, the melting peaks of PLLA-D, PDLA-D, and LD-D were significantly lower than those of the other samples, and their melting enthalpy values were significantly higher. These findings indicated that when the L- and D- molecular chains were shortened by degradation, more nuclei were formed, resulting in lower melting points and higher melting enthalpy in PLLA-D, PDLA-D, and LD-D. In other words, once nuclei have formed under specific storage conditions, crystals form under suitable crystallization conditions. Notably, only stereocomplex crystals were formed in LD-D; that is, no homocrystallization occurred in LD-D during the first heating, cooling, and reheating processes. This finding indicated that when the LD-D sample was stored in the vacuum-free desiccator, no homocrystal nucleus formed. It was also found that at higher molecular weights as in LD-R and LD-V, homocrystal formation prevails as in LD-R and LD-V, whereas at lower molecular weights as in LD-D, stereocomplex crystals formation prevails.

**Figure 9:**
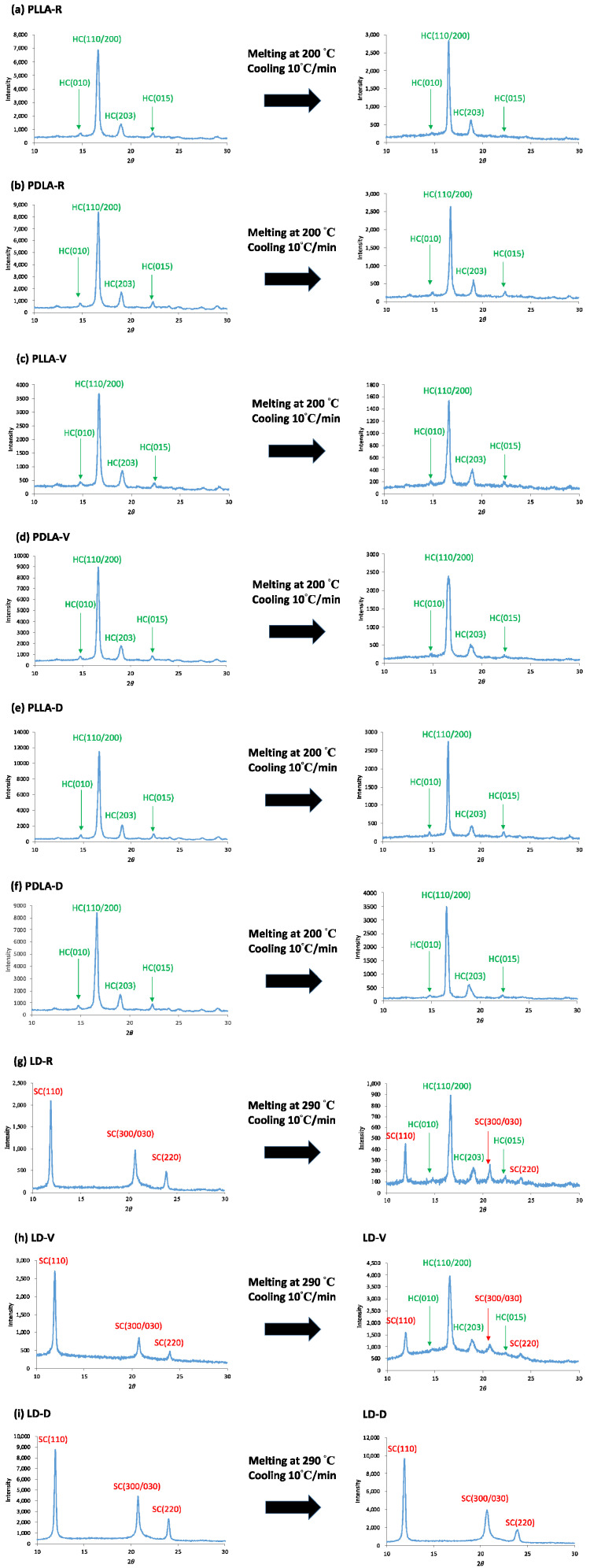
Wide-angle X-ray diffraction (WAXD) profiles of (**a**) PLLA-R, (**b**) PDLA-R, (**c**) PLLA-V, (**d**) PDLA-V, (**e**) PLLA-D, (**f**) PDLA-D, (**g**) LD-R, (**h**) LD-V, and (**i**) LD-D before and after melting and then cooling to room temperature at a rate of 10 °C/min. Here, (010), (110/200), (203), and (015) are related to homocrystal reflections; whereas (110), (300/030), and (220) are related to stereocomplex crystal reflections.

The WAXD profiles of PLLA, PDLA, and LD are shown in Figure 9. PLLA-R, PDLA-R, PLLA-V, PDLA-V, PLLA-D, and PDLA-D all exhibited four diffraction peaks at 14.7°, 16.6°, 19.0°, and 22.3°, which corresponded to the (010), (110/200), (203), and (015) homocrystal reflections, respectively (Figure 9a–f) [28]. After melting at 200 °C for 5 min and then cooling to room temperature at 10 °C/min, these six samples all had identical homocrystal reflection results. PLA is polymorphic, meaning that it depends on crystallization conditions so that different crystal structures may develop. The crystallization of the melt at temperatures higher than 120 °C leads to the formation of orthorhombic α-crystals, in which the molecular segments adopt a 10_3_-helix conformation. At lower crystallization temperatures, the growth of pseudohexagonal α′-crystals is favored, with the molecule segments showing the same helical structure as in α-crystals, but exhibiting conformational disorder [29,30]. Since the WAXD diffraction peaks of the α-crystals and α′-crystals highly overlap, it is difficult to identify separately. In this study, the experimental conditions of PLLA and PDLA were melting at 200 °C and then cooling to room temperature at 10 °C/min. Due to the homocrystallization range of 130–60 °C during cooling (see Figure 7a), both the α-type and α′-type will grow [30,31]. This indicated that homocrystals formed regardless of storage conditions. Here, a heater (THMS 600, Linkam) was equipped with an electric microscope controller (TMS91, Linkam), and was used to prepare the sample preparation. LD-R, LD-V, and LD-D all exhibited three diffraction peaks at 11.9°, 20.7°, and 24.0°, which corresponded to the (110), (300/030), and (220) stereocomplex crystal reflections, respectively (Figure 9g–i) [28]. After melting at 290 °C for 3 min and then cooling to room temperature at 10 °C/min, LD-R and LD-V each exhibited seven diffraction peaks: four at 14.7°, 16.6°, 19.0°, and 22.3°, which were related to homocrystal reflections, and three at 11.9°, 20.7°, and 24.0°, which were related to the stereocomplex crystal reflections [28]. However, among the crystalline diffraction peaks of LD-D before and after melting, only three crystal diffraction peaks related to the stereocomplex crystal were observed; no diffraction peak related to the homocrystal was observed before or after melting. One possible reason for these findings is the strong interaction between the L- and D- molecular chains in LD. When these chains are packed side by side, more stable stereocomplex crystals are formed. The randomly packed L- and D- molecular chains in LD are relatively highly prone to hydrolysis and degradation. Therefore, when these chains are packed side by side to form stereocomplex crystals and randomly arranged L- and D- molecule chains are easily hydrolyzed and degraded, this interferes with the formation of homocrystals in LD. Consequently, the structure of the stereocomplex crystals formed by the L- and D- molecular chains packed side by side is more resistant to hydrolysis than the structure formed by other arrangements of L- and D- molecular chains in the blended sample.

## 4. Conclusions

In this study, PLLA, PDLA, and LD were stored under two sets of laboratory conditions for 3 years to investigate the thermal stability and crystallization behaviors of homocrystals and stereocomplex crystals. The thermal stabilities of PLLA, PDLA and LD stored under two sets of storage conditions are very different. When samples are stored in vacuum-sealed bags, they will degrade slowly; however, when samples are stored in a vacuum-free desiccator, they will degrade significantly. The GPC results of *M*_n_, *M*_w_, and I.V. revealed that the PLLA, PDLA, and LD samples hydrolyzed slowly when stored in vacuum-sealed bags and hydrolyzed significantly when stored in a vacuum-free desiccator. These processes resulted in considerably reduced thermal stability in the PLLA, PDLA, and LD stored in a vacuum-free desiccator.

Compared with the crystallization and melting behaviors of the received PLLA, PDLA, and as-blended LD samples, no significant differences were observed in the melting points or melting enthalpy values of the corresponding samples stored in the vacuum-sealed bags. However, storing the samples in a vacuum-free desiccator caused significant hydrolytic degradation. When the L- and D- molecular chains were shortened by hydrolysis, more nuclei were formed, and this caused the melting points of the PLLA, PDLA, and LD samples to decrease and the melting enthalpy values of the crystals in these samples to increase.

WAXD analysis revealed that only stereocomplex crystals formed in LD-D; no homocrystals were observed. This finding indicated that L- and D- molecular chains packed side by side can form a relatively stable stereocomplex crystal. However, the L- and D- molecular chains randomly packed in LD are highly prone to hydrolysis and degradation. When L- and D- molecular chains are packed side by side the formed stereocomplex crystals, and randomly arranged L- and D- molecular chains are easy hydrolyzed and degraded, which interferes with the formation of homocrystals in LD.

Consequently, in this study, it was found that under two sets of laboratory storage conditions: (1) stored in a vacuum-free desiccator and (2) stored in a vacuum-sealed bag, the hydrolysis levels of PLLA, PDLA and PLLA/PDLA (LD) blends vary greatly. When samples are stored in a vacuum-free desiccator, they will hydrolyze significantly. When the L- and D- molecular chains were shortened by hydrolysis, more nuclei were formed. Due to the strong interaction which occurred between the side-by-side packed L- and D- molecular chains, stereocomplex crystals formed that were more resistant to hydrolysis than were the other arrangements of L- and D- molecular chains in the blended samples. When the LD sample was stored in a vacuum-sealed bag at room temperature for 3 years, only stereocomplex crystals formed, and no homocrystals were observed. Therefore, the structure of the material before degradation will affect the thermal stability and crystallization behavior of the material after degradation.
